# Responses of Soil Quality and Microbial Community Composition to Vegetation Restoration in Tropical Coastal Forests

**DOI:** 10.3390/biology14091120

**Published:** 2025-08-24

**Authors:** Yuanqi Chen, Feifeng Zhang, Jianbo Cao, Tong Liu, Yu Zhang

**Affiliations:** 1 Institute of Geographic Environment and Carbon Peak & Neutrality, School of Earth Sciences and Spatial Information Engineering, Hunan University of Science and Technology, Xiangtan 411201, China; chenyq2016@163.com (Y.C.);; 2 Fujian Provincial Key Laboratory of the Development and Utilization of Bamboo Resources, Sanming University, Sanming 365000, China; caojianbo612@163.com; 3 School of Earth Sciences, Yunnan University, Kunming 650500, China; 4 Hunan Province Key Laboratory of Economic Crops Genetic Improvement and Integrated Utilization, School of Life and Health Sciences, Hunan University of Science and Technology, Xiangtan 411201, China

**Keywords:** soil restoration, forest ecosystem, afforestation, vegetation types, forest conservation, short rotation

## Abstract

Afforestation substantially promotes vegetation restoration and modifies soil physical, chemical, and biological properties. The integrated effects of soil properties on soil quality, expressed via a composite soil quality index (SQI), remain unclear despite variations among individual properties. Here, five vegetation restoration treatments were selected as follows: (1) barren land (BL, control), (2) disturbed short-rotation *Eucalyptus* plantation (REP); (3) undisturbed long-term *Eucalyptus* plantation (UEP); (4) mixed native-species plantation (MF); and (5) natural forest (NF) following >50 years of restoration. Soil physicochemical properties and microbial community compositions were investigated, and soil quality was evaluated by an integrated SQI. Our results showed that vegetation restoration had strong effects on soil physicochemical properties, soil quality, and microbial communities. Most of the soil physicochemical properties exhibited significant differences among treatments. Soil dissolved organic carbon, total nitrogen, and ammonium nitrogen were the three key soil quality indicators. The SQI increased significantly with vegetation recovery intensity. In both UEP and MF, it reached levels comparable to NF, and was higher in UEP than in REP, implying that short-rotation practices impede soil restoration. In addition, microbial biomass (bacteria, fungi, arbuscular mycorrhizal fungi, actinomycetes, and total microbe PLFAs) increased from BL to NF. All plantations exhibited lower microbial biomass than NF, revealing incomplete recovery and a greater sensitivity to soil physicochemical properties. Conversely, the fungi-to-bacteria biomass ratio decreased sequentially (REP > BL > UEP > MF > NF). Strong positive correlations between microbial biomass and the SQI were observed. These results collectively indicate that afforestation with mixed tree species is optimal for rapid soil restoration, and undisturbed long-term monocultures can achieve similar outcomes. These findings highlight that tree species mixtures and reducing disturbance should be taken into consideration when restoring degraded ecosystems in the tropics.

## Simple Summary

Assessing post-afforestation soil quality can identify the most effective vegetation restoration approaches, which is critical to the sustainable management of forest ecosystems. In this study, we evaluated how different vegetation restoration strategies (barren land control, disturbed short-rotation and undisturbed long-term *Eucalyptus* monocultures, a mixed native-species plantation, and a natural forest) affect soil quality and microbial communities in tropical ecosystems. The results showed that vegetation restoration significantly improved soil physicochemical properties and the overall soil quality index (SQI). Crucially, the SQI in the undisturbed long-term *Eucalyptus* monoculture and the mixed native-species plantation reached levels comparable to the natural forest, demonstrating the recovery potential of well-managed plantations. Microbial biomass (bacteria, fungi, arbuscular mycorrhizal fungi, and actinomycetes) increased from barren land to natural forest but remained lower in all plantations than in the natural forest, indicating incomplete microbial recovery. Strong positive correlations existed between microbial biomass and the SQI. The results indicate that intensive disturbances impede soil and microbial recovery, while microbial communities prove to be more sensitive restoration indicators than physicochemical properties alone. Collectively, afforestation with mixed native species offers rapid soil restoration, and undisturbed long-term monocultures can achieve similar soil quality outcomes over time. This work provides critical insights for optimizing tropical and subtropical afforestation practices.

## 1. Introduction

Anthropogenic activities such as deforestation have led to a severe degradation of forest ecosystems, resulting in widespread soil erosion, nutrient depletion, reduced productivity, and biodiversity loss [1,2]. In response, extensive ecological restoration projects have been implemented in recent decades to rehabilitate degraded terrestrial ecosystems [3,4]. These efforts have achieved notable success, especially in enhancing environmental quality and reinforcing carbon sequestration [5,6]. Among the key restoration strategies, afforestation has played a pivotal role in reversing ecosystem degradation, significantly promoting vegetation recovery and, in turn, modifying soil physical, chemical, and biological properties [7]. Conversely, these soil characteristics are intrinsically linked to plant productivity and ecosystem services [8,9,10]. Therefore, assessing post-afforestation soil quality has emerged as a critical component in ensuring the sustainable management of forest ecosystems.

Afforestation approaches modulate vegetation establishment trajectories and soil restoration efficacy in degraded ecosystems via plant–soil feedback loops [11]. Different afforestation strategies, such as monoculture, mixed-species planting, or natural regeneration, can lead to markedly divergent outcomes in soil quality due to variations in tree species composition and their functional traits [12]. Specifically, tree species richness and diversity influence nutrient cycling, rhizosphere chemical properties, mycorrhizal associations, and microbial community dynamics, all of which collectively shape soil restoration trajectories [13,14,15]. In recent years, numerous studies have been conducted to evaluate the effects of afforestation on soil properties, and significant improvements in ecosystem functionality have been frequently recorded. Vegetation restoration enhances soil nutrient cycling and enzyme activities, particularly in degraded landscapes [16]. In southern China’s erosion-prone regions, afforestation can also elevate soil fertility and increase the abundance and diversity of the soil bacterial community [16]. Based on the long-term monitoring in Ghana, it was found that both plantations and secondary forests exhibited soil carbon storage and key physicochemical properties comparable to those of primary forests in analogous climatic zones [17]. However, the outcome of afforestation is not universally positive, and contrasting effects have been documented. For example, the bacterial biomass represented by phospholipid fatty acids (PLFAs) declined with the successional stage [18]. Similarly, in the dry–hot valley of China, afforestation with *Eucalyptus camaldulensis* reduced the population of fungi and total microbial community, urease activity, and the soil quality index [19]. These discrepancies highlight that the effects of afforestation on soil properties are influenced by plant species composition, soil type, and environmental conditions, and different soil properties could produce varied responses [20,21]. For instance, soil total nitrogen content was the highest in coniferous-mixed plantations, but total phosphorus content was the highest in broad-leaved mixed plantations [22]. A meta-analysis concluded that afforestation increased soil carbon and nitrogen but not phosphorus accumulation [23]. Thus, a comparative analysis of soil quality under different afforestation strategies is essential, as it can not only identify the most effective vegetation restoration approaches but also refine soil management frameworks to maximize soil health and ecological functions [24].

Plantations in subtropical and tropical regions are predominantly monocultures dominated by a single tree species. Among these, eucalyptus (*Eucalyptus* spp.) has become a primary plantation due to its fast growth and short rotation (5–7 years), now covering over 5.40 million hectares across subtropical and tropical China [25]. However, successive planting generations under intensive management have caused severe soil degradation and productivity decline [26]. To mitigate these issues, both extending rotation periods and reducing management intensity are proposed as forest management practices [25,27]. Such measures may enhance biodiversity [28,29] and subsequently improve carbon sequestration and soil nutrient retention [27]. Nevertheless, the mechanistic effects of these adjustments on soil physicochemical and biological properties remain poorly resolved. Concurrently, multi-species afforestation has been advocated for superior soil fertility preservation [30]. Moreover, increasing the diversity of plantations is also a promising approach to adapt forests to climate change, which can be a viable and economically accessible strategy for sustainable wood production and reconciling economic and environmental benefits [31,32]. Some studies reported significantly greater soil quality improvements in mixed species plantations compared to monocultures [15,24], yet others documented negligible differences between monocultures and mixed plantations [33,34]. This contradiction underscores the idea that the context-dependent efficacy of forest management strategies on soil rehabilitation remains inadequately quantified.

The soil quality index (SQI) is regarded as an essential instrument for assessing changes in soil quality [35]. The utilization of the SQI can overcome the complexity of soil assessment [36]. To evaluate soil quality changes following vegetation restoration, a chrono sequence study incorporating five distinct land-use types was conducted, which included a barren land (as control), two pure *Eucalyptus* plantations over 50 years (one has undergone a short rotation every 5–7 years and the other is undisturbed), a mixed native-species plantation over 50 years, and a nearby undisturbed natural forest (over 200 years). We hypothesized the following: (1) Vegetation restoration will significantly improve soil quality across all forested sites relative to the barren land, primarily through enhanced soil fertility and microbial abundance, and the mixed native-species forest will have the highest soil quality. (2) Soil quality in the undisturbed long-term *Eucalyptus* plantation will exceed that of the disturbed short-rotation *Eucalyptus* plantation due to reduced management intensity. This work will advance the mechanistic understanding of soil restoration and provide critical guidelines for restoring degraded ecosystems.

## 2. Materials and Methods

### 2.1. Site Description

The study was conducted at the Xiaoliang Research Station of Tropical Coastal Ecosystems, Chinese Academy of Sciences (110°54′ E, 21°27′ N), located in Maoming city of Guangdong Province, China. This region has a typical tropical monsoon climate with a mean annual precipitation of 2000 mm and a mean annual temperature of 23 °C. The soil is classified as a granite-derived Latosol that has been experiencing heavy erosion since the 1950s under harsh hydrothermal conditions [37]. In the degraded barren land, the monthly mean soil temperature at a 0–20 cm depth peaked at 47.5 °C, whereas soil total organic carbon and total nitrogen contents were only 6.0 g kg^−1^ and 0.3 g kg^−1^, respectively [38]. Historically, the native vegetation in this region was evergreen broad-leaved seasonal rainforests. However, extensive deforestation has occurred [38]. The remaining native forests at the site are classified as tropical secondary forests, which have been preserved for over 200 years. Dominating tree species include *Cinnamomum camphora*, *Sterculia lanceolate*, and *Cryptocarya chinensis* [39].

Afforestation practices on the barren land (BL) have been implemented since 1959, although the harsh habitat severely limited natural vegetation recovery. Approximately 3.7 ha of BL was selected and assigned as a control representing the baseline condition prior to restoration. Due to complete topsoil erosion, only few herbaceous plants or xeric shrubs (e.g., *Dicranopteris linearis* and *Eriachne pallescens*) are to be found in the control areas [38]. *Eucalyptus exserta* were planted on the other BL in the early 1960s. Management subsequently diverged: half of the *E. exserta* plantation underwent short-rotation harvesting every 5–8 years (short-rotation *Eucalyptus* plantation, REP), while the other half remained undisturbed since planting (undisturbed *Eucalyptus* plantation, UEP). In 1974, one distinct catchment was clear-cut and reforested with multiple native tree species to create a mixed forest (MF). The vegetation surveys performed in 2015 showed that this MF supported an average of 14.6 native tree species in each 400 m^2^ quadrate, and the dominant tree species are *Aphanamixis polystachya*, *Schefflera octophylla*, *Carallia brachiate*, *Symplocos chunii*, *Acacia auriculaeformis*, *Photinia benthamiana*, and *Cinnamomum burmannii* [39]. This MF stand is developing structural and compositional similarity to the undisturbed secondary natural forest (NF) [38]. Our study includes sites that experienced one of five restoration treatments: (1) barren land (control, BL), (2) disturbed short-rotation *Eucalyptus* plantation (REP), (3) undisturbed long-term *Eucalyptus* plantation (UEP), (4) mixed native-species plantation (MF), and (5) natural forest (NF). The detailed information about soil and vegetation was provided in previous studies [37,38]. Four replicate sampling plots per treatment were established, randomly located at a distance of >20 m apart from each other.

### 2.2. Soil Sampling and Physicochemical Properties

Surface soil samples (0–15 cm depths) were collected in May 2014 from all five vegetation restoration treatments. Within each replicate plot, soils were sampled from five randomly selected microsites using a stainless-steel core (3.0 cm diameter). Visible plant residues and roots were manually removed. The composite soil samples were sieved by a sieve with a 2 mm bore diameter and divided into two subsamples: one was preserved in a field-moist state for immediate analysis of soil moisture content and related parameters, while the other was air-dried for the subsequent determination of soil organic carbon and its associated physicochemical properties.

Soil moisture content (SMC) was measured by oven-drying at 105 °C to constant weight. Soil pH was measured potentiometrically at a soil-to-water ratio of 1:2.5 (*w*/*v*). Soil organic carbon (SOC) was determined by the traditional wet oxidation with potassium dichromate method [40]. Soil total nitrogen (total N) was analyzed by the micro-Kjedahl digestion method, and soil total phosphorus (total P) was digested with a sulfuric acid solution and quantified by the molybdenum–antimony (Mo-Sb) anti-spectrophotometer method. Soil dissolved organic carbon (DOC) was extracted with 0.5 M K_2_SO_4_, filtered (0.45 μm), and analyzed at a high temperature on a TOC analyzer (TOC-VCSH, Shimadzu, Japan). Soil NH_4_^+^-N and NO_3_^−^-N were measured by a flow injection analyzer (AA3, Bran Luebbe) [41].

### 2.3. Soil Microbial Biomass and Community Composition

Soil microbial biomass C (MBC) and N (MBN) were determined by the chloroform fumigation–extraction method [42]. Phospholipid fatty acid (PLFA) analysis was applied to characterize soil microbial community composition, and concentrations of individual PLFAs were quantified based on the internal standard concentration of 19:0 methylester [43]. The PLFAs i14:0, i15:0, a15:0, i16:0, a16:0, i17:0, a17:0, a18:0, i18:0, a19:0, 16:1ω7c, 16:1ω9c, 17:1ω8c, 18:1ω7, cy17:0, and cy19:0 [44] were used as bacterial (B) biomarkers. The PLFAs 18:1ω9c, 18:2ω6,9c [44], and 18:3ω6,9,12c were applied to denote fungal (F) biomarkers. The PLFA 16:1ω5c was considered as an arbuscular mycorrhizal fungal (AMF) biomarker [45]. The PLFAs 10Me 16:0, 10Me 17:0, and 10Me 18:0 were used as actinomycetes biomarkers. Total microbial biomass was represented by the sum of identified bacterial, fungal, AMF, and actinomycetes PLFAs. Soil microbial community structure represented by the fungal-to-bacterial ratio (F:B ratio) was calculated as the sum of fungal biomarker PLFAs divided by the sum of bacterial biomarker PLFAs [44].

### 2.4. Soil Quality Index (SQI) Evaluation

Twelve soil physicochemical and biological properties (SMC, pH, SOC, TN, TP, DOC, DN, NH_4_^+^-N, NO_3_^−^-N, MBC, MBN, and MBC/MBN) were evaluated to identify a minimum dataset (MDS) for soil quality assessment. We employed principal component analysis (PCA) followed by Pearson’s correlation analysis to select the most suitable indicators.

MDS selection procedure: According to Andrews et al. [46], to be MDS potentials, principal components (PCs) must have eigenvalues not less than 1.0 that explain more than 5% of the total variation. Within each retained principal component, those with an absolute value within 10% of the highest loading factor were selected as the important indicators. In addition, if multiple indicators were retained within a single PC and exhibited pairwise Pearson correlation coefficients > |0.6|, the indicator with the smallest absolute loading value in that PC was removed [47].

Scoring and SQI calculation: After MDS indicators were selected, a nonlinear scoring function was employed to convert the soil indicators into scores ranging from 0 to 1. Equation (1) for the soil indicator score was given in Andrews et al. [46]:

S = 1/[1 + (X/X_0_)^b^] (1)

where S is the indicator score, X is the value of the soil indicator, X_0_ is the mean value of each indicator, and b is the value of the equation’s slope. Slope values (b) of −2.5 and 2.5 were used to illustrate a ‘more is better’ and a ‘less is better’ curve, respectively [47,48]. After scoring and weighting all MDS indicators, the integrated SQI was calculated by Equation (2) as follows:

(2) $$\mathrm{SQI}\;=\;\sum_{i=1}^{n}S_{i}\times W_{i}$$

where *S_i_* is the score of the selected indicators, *W_i_* is the weighting of the selected indicators, and *n* is the number of selected indicators [49].

### 2.5. Data Analysis

SMC, soil pH, the concentrations of SOC, TN, TP, NH_4_^+^-N, NO_3_^−^-N, DOC, MBC, MBN, and microbial PLFAs, and the SQI were analyzed by a one-way ANOVA, and then a multiple comparison analysis (LSD) was employed to test the difference between vegetation restoration treatments. A Pearson correlation analysis was applied to test the relationships between the SQI on soil microbial biomass and community structure. Data were reciprocally or square-root-transformed when required to meet the assumptions of normality and homogeneity of variance. Statistical significance was determined at *p* < 0.05. All analyses were performed with SPSS 18.0 software (SPSS Inc., Chicago, IL, USA).

## 3. Results

### 3.1. Soil Physicochemical Properties

Vegetation restoration treatments significantly affected soil physicochemical properties (Table 1). Afforestation on barren land (BL) significantly increased SMC except REP. The SMC was highest in NF, followed by the MF and UEP, all of which were significantly greater than those in BL and REP (*p* < 0.01). Soil pH exhibited an inverse trend, with the highest value in BL and the lowest in NF. The differences between REP, UEP, and MF were not significant (*p* > 0.05; Table 1). SOC concentration increased progressively with restoration intensity (*p* < 0.01). SOC rose significantly from 3.0 g kg^−1^ in BL to 20.5 g kg^−1^ in MF. However, in MF, it remained significantly lower than in NF (26.7 g kg^−1^; *p* < 0.01). SOC concentration in UEP (19.0 g kg^−1^) was nearly double that of REP (9.8 g kg^−1^) and statistically equivalent to MF (*p* = 0.28), and lower than in NF (*p* < 0.01). Interestingly, soil total N recovery varied notably: in MF, it exceeded that of NF (*p* = 0.03), while in UEP, it returned to levels comparable to NF (*p* = 0.32). Soil total N in REP was marginally lower than UEP (*p* = 0.25) but showed no significant difference from that in BL (*p* = 0.73). Soil total P mirrored the response pattern of soil total N to vegetation restoration treatments (Table 1). Soil DOC exhibited similar patterns to SMC, increasing from 159 mg kg^−1^ (BL) to 884 mg kg^−1^ (NF). DOC in REP (181 mg kg^−1^) did not differ significantly from BL (*p* = 0.72). The soil N availability components showed treatment-specific responses. For instance, NH_4_^+^-N was the highest in UEP (*p* <0.01), with no significant differences among BL, REP, MF, and NF (*p* > 0.05), while NO_3_^−^-N peaked in NF and it was significantly higher than the other four treatments (*p* <0.01). The NO_3_^−^-N in UEP was significantly higher than REP (*p* = 0.02).

### 3.2. Soil Microbial Properties

**Figure 1:**
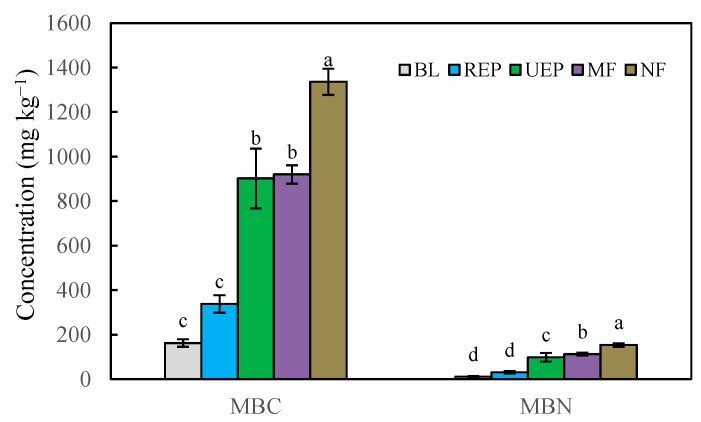
Soil microbial biomass carbon (MBC) and nitrogen (MBN) concentrations in all treatments. BL, REP, UEP, MF, and NF stand for barren land, short-rotation *Eucalyptus* plantation, undisturbed *Eucalyptus* plantation, mixed native-species plantation forest, and natural forest, respectively. Values are means ± SE; n = 4 plots. Different lowercase letters indicate significant differences between different restoration treatments at *p* < 0.05.

Restoration treatments enhanced MBC and MBN (Figure 1). Both MBC and MBN reached peak concentrations in NF, confirming that all restoration treatments facilitated microbial recovery. Specifically, MBC was significantly higher in UEP and MF than in BL and REP (*p* < 0.05), and no significant difference occurred between UEP and MF. Also, MBC in REP remained statistically indistinguishable from BL. For MBN, it was significantly higher in MF than in UEP, and UEP was higher than in REP and BL. There was no significant difference between REP and BL (Figure 1).

**Figure 2:**
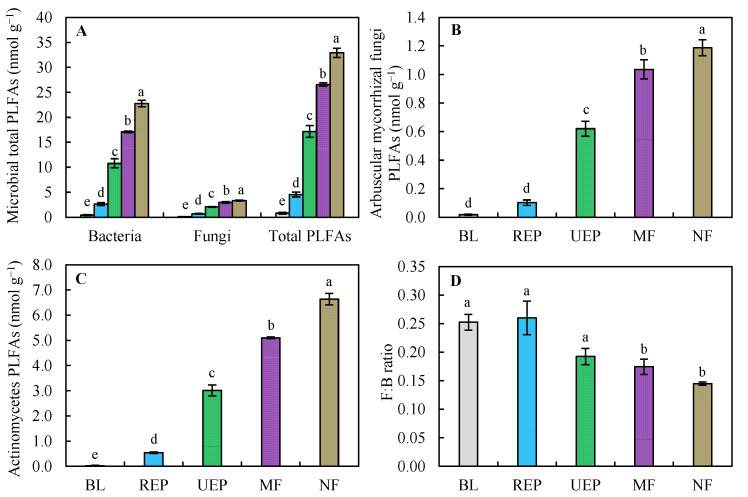
Soil microbial biomass represented by PLFAs in all treatments: (**A**) soil total microbial PLFAs; (**B**) arbuscular mycorrhizal fungi (AMF) PLFAs; (**C**) actinomycetes (Act) PLFAs; (**D**) fungi-to-bacteria ratio in all treatments. Values are means ± SE; n = 4 plots. Different lowercase letters indicate significant differences in the same microbial groups between different restoration treatments at the *p* < 0.05 level. See Figure 1 for abbreviations.

Total microbial biomass (summed PLFAs) and individual biomarker groups (bacteria, fungi, AMF, and actinomycetes PLFAs) increased with vegetation recovery treatments. They were the lowest in BL and the highest in NF for the biomass of bacteria, fungi, total microbes, AMF, and actinomycetes (Figure 2A,C). This meant that microbial biomass in the studied plantations did not reach the NF level. Microbial biomass (bacterial, fungal, total, AMF, and actinomycetes PLFAs) was significantly higher in MF than in UEP, and UEP was significantly higher than in REP. Meanwhile, the microbial biomass in REP was significantly higher than in BL, except for AMF biomass (Figure 2B). Conversely, the fungi-to-bacteria ratio (F:B) decreased along the restoration gradient: BL ≈ REP > UEP > MF > NF. Specifically, F:B ratios in BL, REP, and UEP exceeded those in MF and NF (*p* < 0.05), with no differences among BL, REP, and UEP. The F:B ratio in MF was marginally higher than in NF (Figure 2D).

### 3.3. Soil Quality Index and Its Relationships with Microbial Community

**Figure 3:**
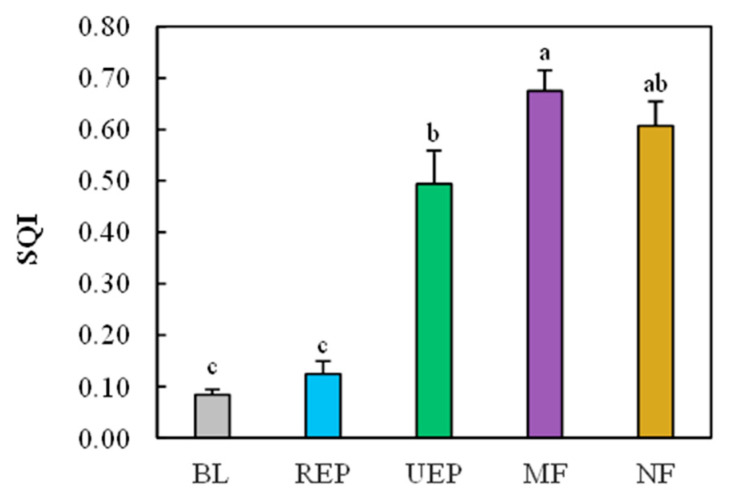
Soil quality index (SQI) for all treatments. BL, REP, UEP, MF, and NF stand for barren land, short-rotation *Eucalyptus* plantation, undisturbed *Eucalyptus* plantation, mixed native-species plantation forest, and natural forest, respectively. Values are means ± SE; n = 4 plots. Different lowercase letters indicate significant differences between different restoration treatments at *p* < 0.05.

PCA yielded three significant components (eigenvalues ≥ 1.0) explaining 89.2% of the total variance (Table 2). SMC, SOC, MBC, DOC, MBN, and DN were highly weighted indicators in PC-1 and were also significantly correlated with each other. DOC had the highest PC-1 weighting (0.978), so it was only retained in the MDS. TN and NH_4_^+^-N had the highest weighting in PC-2 and PC-3, respectively, securing their MDS inclusion. Thus, DOC, TN, and NH_4_^+^-N were the three key soil quality indicators and comprised the final MDS. The SQI was calculated using PCA-derived weighting factors. Vegetation restoration treatments promoted soil quality. The SQI increased from 0.05 to 0.66 with vegetation recovery (Figure 3). It was the lowest in BL and the highest in MF. The SQI in MF is equivalent to the NF levels. The SQI increased marginally in the REP compared with BL, and it was significantly higher in UEP than in REP. Meanwhile, the difference in the SQI between UEP and NF was not significant, while in UEP, it was significantly lower than in MF (Figure 3).

Pearson correlation analysis showed that microbial biomass and community composition characteristics, such as bacteria, fungi, AMF, actinomycetes, and the F:B ratio, exhibited strong SQI linkages. Microbial biomass was significantly and positively correlated with the SQI, while the F:B showed a significantly negative correlation with the SQI (Table 3).

## 4. Discussion

### 4.1. Effects of Vegetation Recovery on Soil Quality

Vegetation recovery improves soil quality [50]. Soil quality index increased from barren land to natural forest in this study (Figure 3). Plant biomass generally drives soil improvement [51,52]. In these studied forests, the mixed plantation and natural forest had comparably high aboveground biomass C stocks and root biomass, which were much higher than those in the short-rotation *Eucalyptus* plantation [53]. Unfortunately, plant biomass in the undisturbed *Eucalyptus* plantation was not investigated, but the higher height and larger diameter at breast height were observed relative to the short-rotation *Eucalyptus* plantation. Therefore, the increase in plant biomass could be responsible for the soil quality improvement in these vegetation restoration treatments. However, compared to barren land (no plants), the greater carbon storage of plant biomass in the short-rotation *Eucalyptus* plantation did not result in a higher SQI in this study, which corresponded to 4.0% to 5.1% of those in the mixed forest and natural forest [53]. This finding suggests that plant biomass cannot fully explain the changes in soil quality in this study, which was consistent with other studies [20,54]. A recent study reported that root morphological traits affected soil quality [36], whereas they were absent in this study. Vegetation types also affected soil quality [19]. The deciduous broad-leaved forest had the highest soil quality index, which was higher than the natural forest, and the disturbed forest had the lowest SQI in the Karst areas of southwest China [12]. Furthermore, the minor SQI discrepancy between barren land and the short-rotation *Eucalyptus* plantation could be ascribed partly to their non-significant differences in DOC, TN, and NH_4_^+^-N in this study.

Soil physicochemical properties affect soil quality [36,55], which was supported by the significant correlations between soil physicochemical properties (SMC, pH, SOC, TN, and TP) and the SQI in this study. Ren et al. [52] found that soil organic matter and available P were the primary factors impacting soil quality. The trends in the SQI were the same as TN and TP among different vegetation types. As the TN was one of the three key soil quality indicators calculating the SQI, the correlations between them could be explained by the inclusion in the index, while the weighted value of TN in the SQI was not the greatest among the three key soil quality indicators (Table A1). Thus, we speculated that TN and TP could be the most important factors mediating soil quality.

Soil physicochemical properties showed different responses to vegetation restoration types [56]. The changes in SOC were more sensitive to vegetation recovery than TN and TP, which was consistent with the previous study [53]. High-intensity management like short-rotation cutting did not increase soil N and P [57]; thus, the improvement in soil quality was not significant in our study. Forest conservation or reducing disturbance facilitates the improvement of soil quality in plantations [58]. Brown et al. [17] reported that after 40 years of restoration, the soil carbon stocks and key soil physicochemical properties in plantations and secondary forests reached similar levels to those in the primary forests in the wet and moist climatic zones of Ghana. The significant improvement in soil physicochemical properties under protected management likely enhances soil quality, potentially explaining the SQI discrepancy between undisturbed long-term and disturbed short-rotation *Eucalyptus* plantations observed in this study.

### 4.2. Effects of Vegetation Recovery on Soil Microbial Community

Vegetation recovery significantly increased microbial biomass, regardless of MBC or PLFAs (Figure 1 and Figure 2), aligning with the findings by Zhang et al. [59]. However, microbial communities responded differently. Notably, Zhang et al. [59] found that there was no significant difference in fungal biomass between the mixed forest and natural forest in the topsoil. No significant difference in AMF biomass between the barren land and short-rotation *Eucalyptus* plantation was also detected in the previous study [37], which was in accordance with our study. The shifts in microbial communities could probably be driven by vegetation recovery and the associated changes in soil physicochemical properties [60,61]. The significant correlations between soil physicochemical properties and microbial biomass supported these findings (Table A2). Soil microbial community composition was a more sensitive indicator to reflect the restoration of the ecosystem relative to soil physicochemical properties. Mixed plantations had higher microbial biomass than pure plantations, possibly due to higher biodiversity [62]. The positive effects of plant diversity on microbial biomass were shown across terrestrial ecosystems [63]. Litter as a substrate for microbes may mediate microbial communities [64]. However, there was no significant difference in litter biomass among the short-rotation *Eucalyptus* plantation, mixed forest, and natural forest [39]. Thus, the root-derived inputs rather than litter mass may exert a stronger influence on microbial biomass dynamics.

Elevated microbial biomass may likely contribute to enhanced soil quality through a greater accumulation of microbial residues. Previous studies have found that the accumulation of glomalin-related soil protein and amino sugars accelerated with vegetation recovery [37,65]. This link was further supported by significantly positive correlations between microbial biomass and the SQI (Table 3). The F:B ratio decreased as the restoration process progressed, which was in accordance with a previous study [65,66]. This could be explained by a faster increase in bacteria than fungi. Bacteria were more sensitive to local environmental drivers than fungi in forest ecosystems, particularly soil pH [66].

Beyond microbial biomass, soil microbiota functional recovery was also observed in the same plots with this research. Microbial enzyme activities and soil biodiversity in the mixed plantation reached comparable levels to the natural forest in terms of soil microbes and mite diversity [39,59]. This indicates significantly faster recovery of soil biodiversity under native-species mixed plantations than under disturbed *Eucalyptus* monocultures on these degraded tropical coastal terraces after over 60 years.

For the MDS-selected soil quality indicators, their ability to represent integrated soil quality remains constrained by the absence of key soil physical properties and plant parameters. Furthermore, the unmeasured plant biomass in the UEP hindered a direct assessment of its influence on soil quality. Additionally, although this study indicated comparable soil quality among UPE and NF, *Eucalyptus* monocultures are known to weakly support biodiversity [67], demonstrating lower bird functional diversity in relatively young plantations than in natural forests [68]. It thus raises concerns about broader ecosystem function beyond soil quality alone, necessitating urgent long-term monitoring of undisturbed *Eucalyptus* plantations.

## 5. Conclusions

Vegetation restoration treatments strongly influence soil quality and microbial communities. The SQI in the undisturbed long-term *Eucalyptus* plantation and mixed native-species forest reached levels comparable to the natural forest, indicating the recovery potential of well-managed plantations. Microbial biomass (bacteria, fungi, arbuscular mycorrhizal fungi, and actinomycetes) progressively increased from barren land to natural forest, but remained lower in all plantations than in the natural forest, suggesting incomplete microbial recovery and more sensitive responses relative to soil physicochemical properties alone. In addition, soil dissolved organic carbon, total nitrogen, and NH_4_^+^-N were three key soil quality indicators. Soil microbial biomass positively affected the SQI. Moreover, an undisturbed long-term *Eucalyptus* plantation has higher SQI and microbial communities than a disturbed short-rotation *Eucalyptus* plantation, indicating that intensive disturbance impeded the recovery of soil quality. Collectively, these findings suggest that afforestation with mixed native tree species is more favorable, and long-term forest protection implemented in pure plantations also proves effective for restoring degraded landscapes. These two restoration approaches enable soil quality to approach levels observed in the secondary native forest over time. This study highlights that tree species mixtures and reducing disturbance should be taken into consideration for ecological restoration in tropical and subtropical ecosystems.

## Figures and Tables

**Table 1:** Soil physiochemical properties in five vegetation restoration treatments.

Parameters	BL	REP	UEP	MF	NF
SMC (%)	10.1 ± 0.5 c	9.5 ± 0.3 c	19.6 ± 0.8 b	19.9 ± 1.1 b	23.3 ± 0.4 a
pH value	4.7 ± 0.05 a	4.4 ± 0.02 b	4.4 ± 0.04 bc	4.3 ± 0.1 bc	4.2 ± 0.04 c
SOC (mg g^−1^)	3.0 ± 0.2 d	9.8 ± 0.5 c	19.0 ± 1.6 b	20.5 ± 0.8 b	26.7 ± 0.7 a
TN (mg g^−1^)	0.01 ± 0.01 c	0.09 ± 0.04 c	0.35 ± 0.24 bc	1.16 ± 0.11 a	0.58 ± 0.22 b
TP (mg g^−1^)	0.02 ± 0.01 c	0.01 ± 0.01 c	0.08 ± 0.03 bc	0.16 ± 0.03 a	0.11 ± 0.03 ab
DOC (mg kg^−1^)	159 ± 26 d	181 ± 13 d	407 ± 62 c	694 ± 35 b	884 ± 50 a
NH_4_^+^-N (mg kg^−1^)	4.1 ± 0.6 b	7.0 ± 2.1 b	28.1 ± 7 a	9.0 ± 2.3 b	6.1 ± 1.9 b
NO_3_^−^-N (mg kg^−1^)	3.5 ± 0.3 bc	1.8 ± 0.5 c	4.6 ± 0.5 b	3.7 ± 0.7 bc	11.2 ± 1.4 a

Note: BL, REP, UEP, MF, and NF represent barren land, short-rotation *Eucalyptus* plantation, undisturbed *Eucalyptus* plantation, mixed native-species plantation forest, and natural forest, respectively. SMC, SOC, TN, TP, DOC, NH_4_^+^-N, and NO_3_^−^-N stand for soil moisture content, soil organic carbon, total nitrogen, total phosphorus, dissolved organic carbon, ammonium nitrogen, and nitrate nitrogen, respectively. Values are means ± SE; n = 4 plots. Different lowercase letters indicate significant differences between different restoration treatments at the *p* = 0.05 level.

**Table 2:** Results of principal component analysis (PCA) of soil quality indicators in the 0–15 cm soil layer of the vegetation restoration chronosequence.

Principal Components	PC-1	PC-2	PC-3
Eigenvalues	8.20	1.38	1.13
Variance (%)	68.35	11.46	9.38
Cumulative (%)	68.35	79.82	89.20
Weighting value	0.346	0.34	0.314
Factor loading			
SMC	0.962	−0.093	−0.088
pH	−0.771	0.206	−0.021
SOC	0.968	−0.051	−0.028
TN	0.645	0.734	0.053
TP	0.743	0.630	0.118
MBC	0.967	−0.149	0.017
DOC	0.978	−0.059	0.117
MBN	0.976	−0.039	0.035
DN	0.955	−0.067	0.082
MBC/MBN	−0.682	−0.132	0.410
NH_4_^+^-N	0.260	−0.162	−0.889
NO_3_^−^-N	0.698	−0.556	0.345

Note: SMC, pH, SOC, TN, TP, MBC, DOC, MBN, DN, MBC/MBN, NH_4_^+^-N, and NO_3_^−^-N, stand for soil moisture content, pH value, soil organic carbon, total nitrogen, total phosphorus, microbial biomass carbon, dissolved organic carbon, microbial biomass nitrogen, dissolved nitrogen, ratio of microbial biomass carbon to nitrogen, ammonium nitrogen, and nitrate nitrogen, respectively.

**Table 3:** Pearson correlation coefficients (r) indicating the direction and strength of relationships between soil physicochemical properties (microbial biomass) and the soil quality index (SQI) (** *p* <0.01, *** *p* < 0.001).

Parameter	SMC (B)	pH (F)	SOC (TMB)	TN (Act)	TP (AMF)	-- (F:B)
SQI	0.94 ***	−0.68 **	0.89 ***	0.75 ***	0.79 ***	--
	(0.89 ***)	(0.92 ***)	(0.90 ***)	(0.90 ***)	(0.91 ***)	(−0.73 ***)

Note: SMC, pH, SOC, TN, TP, B, F, TMB, Act, AMF, and F:B stand for soil moisture content, pH value, soil organic carbon, total nitrogen, total phosphorus, the PLFAs of bacteria, fungi, total microbes, actinomyces, and arbuscular mycorrhizal fungi, and the ratio of fungi to bacterial PLFAs, respectively.

## Data Availability

The data supporting this study’s findings are available from the first author and the corresponding author upon reasonable request.

## Abbreviations

The following abbreviations are used in this manuscript:

SQI	Soil quality index
BL	Barren land
REP	Disturbed short-rotation *Eucalyptus* plantation
UEP	Undisturbed long-term *Eucalyptus* plantation
MF	Mixed native-species plantation
NF	Natural forest
AMF	Arbuscular mycorrhizal fungi

## Appendix A

**Table A1:** Normalization equation of scoring curves.

Parameter	Average	Curve Type	Slope (b)	Normalization Equation	Weighting Value (W)
	(x0)				
DOC	0.40	More is better	−2.5	S = 1/(1 + (X/0.4)^−2.5^)	0.346
TN	452.81	More is better	−2.5	S = 1/(1 + (X/452.81)^−2.5^)	0.340
NH_4_^+^-N	10.75	More is better	−2.5	S = 1/(1 + (X/10.75)^−2.5^)	0.314

Note: DOC, TN, and NH_4_^+^-N stand for soil dissolved organic carbon, total nitrogen, and ammonium nitrogen, respectively.

**Table A2:** Pearson correlation coefficients (r) indicating the direction and strength of relationships between soil physicochemical properties and microbial biomass (* *p* < 0.05; ** *p* <0.01; *** *p* < 0.001).

	SMC	pH	SOC	TN	TP	DOC	DN	NH_4_^+^-N	NO_3_^−^-N
B	0.95 ***	−0.71 **	0.94 ***	0.64 **	0.72 ***	0.97 ***	0.94 ***	0.51	0.74 ***
F	0.94 ***	−0.72 **	0.96 ***	0.69 **	0.74 ***	0.93 ***	0.90 ***	0.30	0.64 **
TPLFAs	0.95 ***	−0.71 **	0.95 ***	0.66 **	0.74 ***	0.96 ***	0.94 ***	0.24	0.71 **
Act	0.94 ***	−0.71 **	0.94 ***	0.67 **	0.75 ***	0.96 ***	0.95 ***	0.22	0.76 ***
AMF	0.94 **	−0.65 **	0.93 ***	0.71 **	0.77 ***	0.94 ***	0.91 ***	0.25	0.66 **
F:B	−0.83 ***	0.56 *	−0.76	−0.52 *	−0.63 **	−0.84 ***	−0.85 ***	−0.20	−0.65 **

Note: SMC, pH, SOC, TN, TP, DOC, DN, NH_4_^+^-N, NO_3_^−^-N, B, F, TPLFAs, Act, AMF, and F:B stand for soil moisture content, pH value, soil organic carbon, total nitrogen, total phosphorus, dissolved organic carbon, dissolved nitrogen, ammonium nitrogen, and nitrate nitrogen, the PLFAs of bacteria, fungi, total microbes, actinomyces, and arbuscular mycorrhizal fungi, and the ratio of fungi to bacterial PLFAs, respectively.
